# Supplementary material for: Chromothripsis during telomere crisis is independent of NHEJ, and consistent with a replicative origin
Source: Genome Res. 2019 May;29(5):737–49. doi: 10.1101/gr.240705.118 (PMC6499312; doi:10.1101/gr.240705.118)
Supplement: Supplemental Material [file supp_gr.240705.118_Supplemental_file_1.zip › contigs/annotated_contigs/DB48/contig.2.DB48_length_840_mean_cov_13.144047619.docx]

**DB48_length_840_mean_cov_13.144047619**

```
ATGCGCATGTTATAGACTGGAATGTGTTACCTTCAGAAACCAAACCTTGACGGGTATGTATATGT|ACAATGATCTTGGATAAACTTTG
                                                                  >chr7:51673249-5167365
AAGGCATAATCATGAAAATCCATTAATGGAAGTTCATTTTCCCTAATCCTCTCCCTTATTTGAAAATCTCTCTTTCTGTAAAGGTACTT
9 + E=9e-235
TGTGTTCTCCTTTAGCAGGTGTAGTCCCCCGTGTACACGATTCCGTCTCAGGACTCACACCCATGCTGTGATAATTTATTAACGAAGAA

CTTATGCATAGTTCACTGCAGGTTGTGAGAGTGAGCATCGTCTGGTTTATGTTTTTGTCTTCGTTGCCCACAGCCGTACCTGGTACCTA

GTAGTCAGTAATAAATGGATGAACAGGGAGTGAGTAACAGGCACACTGGTTCTCAGCTCTGGCCGCACTTTAGAGTCCTCTGGGGAGCT

TTTATATAATATTGATGCCTGAAAACT|TCCC|ACTTCTATTGTATAAAATCAATAAAAACATTACAGCCAGCCCTCTGTAGCCATGAG
                            >chr7:51677489-51677858 - E=4e-204
TTTCACATTCACAGATTCAATCAATGGTTGATGAAAAACTTTTGGGAAAAATATCAATAAAAAATAAAAATACAACAATAAAAAATAGT

GCAAATAAAAAATATAGTATATCAACTTTTTACATAGCAATGACATTGTATCAGGTATCAATAATTAACTTAGAAATGACTGAAAGCAT

ATGGGAGAATGTGCATAGGTCATATGAAAATAACGCACCATTTTGTATCAAGGACTTGAGCTTCCTTGGATTTCAGTACCCACAGGGTG

TCCTGGAACCAATCCCCCATGAATACTGAGGGAAGACTGTAT
```
